# Local

**Published:** 1857-10

**Authors:** 


### LOCAL.

In a previous number of our Journal, we suggested to our country exchanges the propriety and profit of making their columns more original, by industriously gathering items from the oldest inhabitants as to occurrences and places of bygone times. *The New York Observer*, of July 23, embodies the same idea in its application to Fourth of July Orations, its importance and utility, thus:

"On the Fourth of July last, the Rev. Mr. Tuttle, of Rockaway, N. Y., delivered an oration at Madison, to which we now refer, for the purpose of suggesting a hint to future orators for such occasions. Mr. T. had gathered, from written history and personal conversation with the oldest inhabitants, all the facts that could be collected respecting the incidents of the American Revolution, as they occurred on that ground and in its vicinity. Morris County is, indeed, specially rich in such materials; and Mr. T. had, therefore, great facilities for making an oration of interest to the descendants of those whose names and deeds he commemorated. But almost every part of our country, within the thirteen original States, would furnish incidents of value that ought to be preserved in the written records of American literature; and thousands of these incidents being as yet unwritten, will lose their authority when the survivors of revolutionary times shall have passed away. It is, therefore, greatly to be desired, that gentlemen, in preparing orations for the Fourth of July, should interest themselves and their hearers in collecting and reciting those local historical facts, which make so many of our valleys and hill tops classic and sacred. By such efforts, a vast amount of valuable material for future history would be rescued from oblivion."

